# Body Condition Score and Milk Production on Conception Rate of Cows under a Small-Scale Dairy System

**DOI:** 10.3390/ani9100800

**Published:** 2019-10-14

**Authors:** Luis Javier Montiel-Olguín, Felipe J. Ruiz-López, Miguel Mellado, Eliab Estrada-Cortés, Sergio Gómez-Rosales, Juana Elizabeth Elton-Puente, Hector Raymundo Vera-Avila

**Affiliations:** 1 Centro Nacional de Investigación Disciplinaria en Fisiología y Mejoramiento Animal-INIFAP, km.1 Carretera a Colón, Ajuchitlán, Colón, Querétaro 76280, Mexico; montiel.luis@inifap.gob.mx (L.J.M.-O.); ruiz.felipe@inifap.gob.mx (F.J.R.-L.); gomez.sergio@inifap.gob.mx (S.G.-R.); 2 Facultad de Ciencias Naturales, Universidad Autónoma de Querétaro, Av. de las Ciencias s/n, Santiago de Querétaro, Querétaro 76230, Mexico; elizabeth.elton@uaq.edu.mx; 3 Departamento de Nutrición Animal, Universidad Autónoma Agraria Antonio Narro, Calzada Antonio Narro 1923, Buenavista, Saltillo, Coahuila 25315, Mexico; mmellbosq@yahoo.com; 4 Campo Experimental Centro Altos de Jalisco-INIFAP, Av. Biodiversidad 2470. Tepatitlán de Morelos, Jalisco 47600, Mexico; estrada.eliab@inifap.gob.mx

**Keywords:** reproductive performance, dairy cattle, lactation number, fertility, Holstein

## Abstract

**Simple Summary:**

Small-scale dairy farms are important because they generate jobs in rural areas and improve food security, income, and livelihood worldwide. On the other hand, high fertility is strongly associated with the profitability of dairy farms. Therefore, improving reproductive performance would increase milk production and farmers’ incomes. In the present study, we focus on the effect of body condition score (BCS) of Holstein cows at calving and when they are bred for the first time (after calving) on a key reproductive performance indicator: conception rate to first service. Additionally, we studied the association between BCS at calving and milk production, assuming that cows had a moderate genetic potential for milk production. Our results highlight the importance of BCS on the future reproductive performance and productivity of cows in a small-scale dairy system. These results are useful to implement management strategies in order to increase conception rate to first service and milk production in this type of dairy farm.

**Abstract:**

Management and production characteristics impact conception rate to first service (CR1S) in small-scale dairy farms, but the impact of body condition score (BCS) and milk production levels on cows’ fertility is unknown. Our objective is to determine the effect of BCS and milk production on CR1S in small-scale dairy farms of western Mexico. Logistic regression models are used to determine the effect of BCS (at calving and first service), 60-d and 305-d milk production, protein and fat production, lactation number, and days at first service on CR1S. BCS at calving does not affect CR1S in cows with three or more lactations (39.5%; *p* > 0.1). However, first-lactation cows with BCS < 3.0 at calving and second lactation cows with BCS ≤ 2.5 at calving have higher CR1S (63.2 and 67.9%, respectively; *p* < 0.1). This result is perhaps due to reduced milk production, which leads to lower metabolic stress. BCS ≤ 2.5 at calving is associated (*p* < 0.05) with a reduced milk yield, explaining partially the observed higher CR1S in these groups. Cows with BCS ≤ 2.5 at first service in the higher quartile of 60-d milk production (≥ 28kg/day) show lower CR1S (23.9 and 51.1%, respectively; *p* < 0.01). In conclusion, BCS at calving and at first service, 60-d milk production, and lactation number are factors associated with CR1S.

## 1. Introduction

Improving milk yield in small-scale dairy farms offers possibilities to reduce poverty and improve the incomes and livelihoods for peasants in rural areas [1,2,3]. It is estimated that around 150 million small-scale dairy farms exist worldwide and that approximately 750 million people directly depend on this activity [1]. Additionally, small-scale dairy operations improve food security and the nutrition of rural communities and provide intangible social benefits [4]. Mexico is the 14th largest milk producer in the world and the second in Latin America [5]. Furthermore, small-scale milk production farms approximately contribute 35% to marketed national milk production, own approximately 23% of the cattle inventory, and represent 78% of dairy herds in the country [6,7]. Despite the importance of the small-scale dairy system, these farms operate sub-optimally, which compromises profitability [8,9,10].

First-service conception rate is strongly associated with the profitability of dairy farms [11]. Furthermore, this variable allows one to monitor general reproductive performance, particularly the efficiency and accuracy of estrus detection and proper reproductive management during the postpartum period. In intensive dairy farms, the conception rate to first service is affected by body condition score (BCS) at calving and at first service [12,13], milk production levels [13,14], milk fat and protein content [15], and lactation number [15]. However, small-scale dairy farms in developing countries have different management and productive characteristics; for example, diets usually are inadequate for maximum milk yield [16,17] and, consequently, these farms are characterized by suboptimum milk production [18]. These constraints impact conception rate to first service in these dairy farms, but the impact of these limitations on cows’ fertility has seldom been studied. Determining the impact of these factors on the reproductive efficiency of cows would allow for the implementation of strategies to improve the profitability of these dairy farms. Therefore, the aim of this study is to determine the effect of BCS, milk production and composition, and lactation number and days to first service, on first-service conception rate. An additional objective is to determine the effect of BCS at calving on milk production. It is hypothesized that these factors impact the first-service conception rate and milk production in cows kept in traditional small-scale dairy farms.

## 2. Materials and Methods

### 2.1. Farms Selection, Housing, and Feeding

An observational prospective cohort study was conducted in western Mexico, in Los Altos area (20°49′01″N, 102°43′59″O, 1800 m ASL) in the Jalisco state. This area contributes 19% of total national milk production, being the most important milk production zone in Mexico [19]. Additionally, 60% of milk produced in this region comes from small-scale dairy farms [19]. The weather is temperate and sub-humid, with minimum and maximum temperatures of 4.2 and 31.6 °C, respectively. Twenty-three dairy farms were included in the study based on the fulfillment of four criteria: (1) exclusively family labor, (2) herd size between 10 and 100 milking cows (mean ± SD 53.7 ± 6.2), (3) milk production as the main purpose of the operation, and (4) intermediate machine milking level (few individual milking machines and without cooling systems). Ninety-nine percent of the cows included in the study were Holstein, with an average lactation number, age in months, and kilograms of milk per day of 2.7 ± 0.09, 52.2 ± 4.3, and 22.7 ± 4.7, respectively. Regarding herd structure, cows of first, second, third, and fourth or more lactations represented 28.7, 25.1, 14.7, and 31.5% of cows included in the study, respectively.

Cows included in the study were considered clinically healthy, and the management of farms was not modified during the study period. Cows were housed in open-air, dirt-floor pens, and they had unlimited access to fresh water. Cows were milked twice daily at 05:00 and 17:00, and the average annual milk production per cow was 7350 ± 105 kg. Nutritional management details have been described elsewhere [20], and two groups were identified: total confinement and pasture-based dairy farming. A total mixed ration was offered to confined cows. In general, during the entire lactation, confined cows were fed with diets containing 45% forage and 55% grain on DM basis [20]. It has been reported that the feeding strategies are highly diverse in this region; having diets with concentrate oscillating between 34 and 72% [20], indicating the existence of nutritional management problems. Forages were corn silage (70%) or corn stubble (55%) (DM basis), and different commercial concentrates were used (20% crude protein). Cows were fed ad libitum two times daily at 06:00 and 18:00. Farms with cows on pasture were supplemented with corn stubble or corn silage and commercial concentrates when forage supply was limited. For both groups of cows, management and diets offered were inadequate to fulfill the milk production potential of cows, and were characterized by reduced dry matter intake, incorrect forage:grain ratio, low levels of crude protein, and high levels of NDF and ADF [17,20].

### 2.2. Reproductive Management

All cows were not vaccinated against abortifacient diseases. Herd owners treated fresh cows suffering retained placenta and clinical metritis. Visual observation was used for estrus detection for one hour in the morning and one in the afternoon. Breeding began after 45-days postpartum. Cows in estrus in herds using artificial insemination (AI) were submitted for service following the am–pm guideline. Farms using natural breeding kept the bulls permanently with cows. Conception rate was defined as the number of cows that became pregnant at first service divided by the total number of cows that received the first service. To avoid the confounding factor of early pregnancy loss, pregnancy diagnoses were performed at 55 ± 3 days post service by rectal palpation of the uterine contents.

### 2.3. Data Management

Over the course of 18 months, the following reproductive events were recorded: date of calving and first service, BCS at calving and first service and the outcome of pregnancy diagnosis. Three experienced technicians scored body condition using a scoring system previously suggested [21]. The change in BCS was obtained by subtracting the BCS at calving from BCS at first service. For the analysis of milk production and components, databases of BNIL (National Bank of Dairy Information) were used. These databases are part of the National Genetic Improvement Program financed by federal resources for genetic improvement of cows and to enhance the profitability of dairy farms in Mexico. Variables analyzed were 60-days milk production (60 dim), and the standardized milk production, fat, and protein at 305 days post calving. Data of cows with less than three records were eliminated to obtain a more precise standardized calculation. For the final analysis, 279 lactations were obtained, with complete information for all the variables of interest.

### 2.4. Classification of Variables

A pragmatic categorization by quartiles (Q) was used according to the distributions of the sample [22]. Table 1 shows the limit values for the categories of each variable. Each variable was classified into three categories: Q1, for values equal or lower than the limit of the first quartile; values between Q1 and Q3 (Me); and, Q3, for values equal or greater than the limit of the third quartile, with the rationale of comparing the extreme values of the samples’ distribution [22]. The quartile distributions of the variables BCS at first service and change in BCS allowed one to establish only two categories. BCS at first service was categorized as Q1 for the values equal or lower than the limit of the first quartile and MeQ3 for the values greater than the limit of the first quartile. Change in BCS was categorized as Q1Me for the values lower than the limit of the third quartile and Q3 for the values equal or greater the limit of the third quartile. Lactation number was categorized as 1, 2, 3, and ≥ 4.

### 2.5. Statistical Analysis

All analyses were performed using SAS 9.3 (SAS Institute Inc., Cary, NC, USA). Multiple logistic regression models with non-collinear regressors were used to determine the effect of independent variables on first-service conception rate according to the methodology proposed by Potter et al. [23]. The first step was to perform simple logistic regression models with the LOGISTIC procedure of SAS between study variables and first-service conception rate. The level of significance used to retain variables that would be part of the multiple models was set at *p* ≤ 0.35 [23]. From the simple logistic regression analyzes, odds ratios (OR) were obtained as a measure of association between the study variables and conception rate to first service. Subsequently, to avoid collinearity in multiple models, correlation coefficients were obtained in paired tests between the retained factors with the FREQ procedure option CHISQ of SAS. When the confidence limits of the correlation coefficient did not include 0 (indicating collinearity), both variables were not included in the same multiple model. Finally, to obtain the most parsimonious models, the backward option of the LOGISTIC procedure of SAS was used to eliminate non-significant independent variables and/or interactions at *p* > 0.1 [23]. To determine the effect of BCS at calving on 60-d and 305-d milk production, χ2 tests were used (FREQ procedure CHISQ option of SAS). For this analysis, *p* < 0.05 was the threshold for declaring statistical significance and values *p* < 0.1 were considered as trend indicators.

## 3. Results and Discussion

### 3.1. Impact of Study Variables on First-Service Conception Rate

In the present study, overall first-service conception rate was 46.6% (130/279), ~4% below optimum suggested for this production system [16]. The rationale for this study was that factors influencing first-service conception rate in cows on intensive dairy systems would not have an impact on cows kept in small-scale dairy farms given differences in management conditions and productive characteristics. However, the factors retained to be part of the multiple models were lactation number, BCS at calving, BCS at first service, milk production at 60dim, and 305-d standardized protein (Table 2).

**Figure 1:**
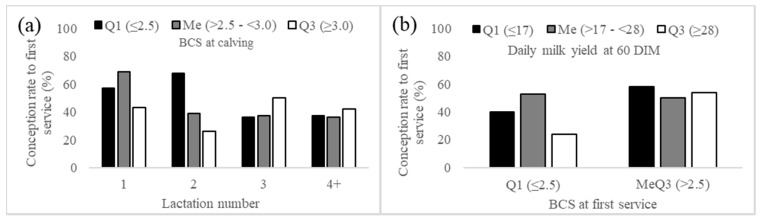
Significant interactions in multiple models of conception rate at first service. (**a**) Significant interaction in model 1, lactation number, and BCS at calving (*p* = 0.096); (**b**) significant interaction in model 2, BCS at first service, and daily milk yield at 60 DIM (*p* = 0.061). MeQ3, values greater than the limit of the first quartile; Q1, values equal or lower than the limit of the first quartile; Me, values between Q1 and Q3; Q3, values equal or greater than the limit of the third quartile.

**Figure 2:**
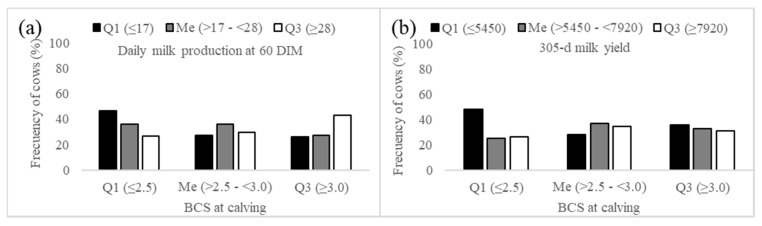
Frequencies at each level of production, according to the BCS at calving on the proportion of cows according to the level of milk production in small-scale dairy farms in western Mexico. (**a**) relationship between BCS at calving and daily milk production at 60 DIM (*p* < 0.05); (**b**) relationship between BCS at calving and 305-d milk yield (*p* = 0.055). Q1, values equal or lower than the limit of the first quartile; Me, values between Q1 and Q3; Q3, values equal or greater than the limit of the third quartile.

Table 3 shows multiple models with non-collinear regressors for first-service conception rate. The results of multiple logistic regression analysis indicated that in model 1, the number of lactation × BCS at calving interaction was significant (*p* = 0.096, Table 4). In model 2, the main effect BCS at first service and the BCS at first service × milk production at 60dim interaction were significant (*p* < 0.1, Table 4). Our results indicate that first-service conception rate was not affected by BCS at calving in cows with three or more lactations (Figure 1a). However, first-lactation cows with BCS at calving < 3.0 and second lactation cows with BCS ≤ 2.5 had higher first-service conception rate. This result seems contradictory; an explanation could be that first and second-lactation cows with low BCS at calving were first serviced later in lactation (105 ± 6 vs. 91 ± 6 dim), which could increase the probability of getting pregnant. However, the calving-first service interval was not statistically significant (Table 2); incidentally, this finding could justify future studies exploring the ideal voluntary waiting period in this type of dairy farms. Another explanation relies on the metabolic stress that generates higher levels of milk production associated with higher parities. First and second-lactation cows with low BCS at calving have limited body energy reserves, which limits milk yield, and this leads to a mild-low negative energy balance [13,23,24]. On the other hand, the loss of BCS is small (Table 2), suggesting that the depth of the negative energy balance in these cows is much lower than that observed in cows in intensive dairy farms [12,13,25]. Therefore, it is reasonable that first and second-lactation cows with lower BCS at calving, without a deep negative energy balance (small loss of BCS) and without the metabolic stress that generates high milk yields (as it occurs in cows with multiple lactations), show higher first-service conception rate [26]. This hypothesis is supported by the finding that 48% of cows in the lower quartile of 60-d milk production calved with BCS ≤ 2.5 (Figure 2a). One factor that was not considered in the design of this study was the genetic merit for milk production. High-yielding cows tend to present lower first-service conception rate [26]. Cows in the present study produced more milk than cows under the same production system in other studies [9,17,18], which would suggest that their genetic merit is slightly higher. Furthermore, it has been published that Mexican Holstein cows in both intensive and small-scale dairy systems share common ancestry bulls (presumably with high genetic merit for milk yield) from USA and Canada [27], supporting the idea of higher genetic merit. However, approximately one-quarter of the services in this type of farms correspond to herd bulls [28], an activity that limits genetic improvement. Although the genetic merit for milk yield of these cows has not been determined yet, this information suggests that the genetic merit of cows in the present study could be higher than other cows under the same production system, but it may not be as high as that in cows in intensive farms (because of the dependence of natural breeding). This assumption (medium-high genetic merit) would explain partially the good overall conception rate at first service observed in the present study (Figure 1). Taken together, these results suggest that lactation number, BCS at calving, and 60-d milk production influence first-service conception rate in cows under this medium-input dairy farms.

In intensive dairy farms, BCS at first service and milk production level negatively affect conception rate at first service [13]. It is worth noting that in the present study, on average the first service occurred far beyond the optimum time suggested for this production system (70 days) [16]. Despite limited nutrient supplies, anestrus postpartum is not a problem in these cows [10,28], presumably as a result of the low negative energy balance (change in BCS) and the suboptimal milk production levels (Table 1). However, in these farms, delayed first services could be a problem associated with deficient estrus detection [10,28]. On the other hand, cows with low BCS at first service (≤ 2.5) in the higher quartile of 60-d milk production (≥ 28 kg) presented lower first-service conception rate. These results were also expected because of the negative association between milk production and first-service conception rate [25,26]. Taken together, this information suggests that conception rate decreased in cows with low BCS at first service and that this effect is exacerbated when individual milk production levels are high for these dairy farms. Consequently, a practical suggestion for increasing conception rate in this production system is to provide the first service to cows with a BCS ≥ 2.75 (regardless of milk production level).

### 3.2. Effect of Body Condition Score on Milk Production

The premise of this analysis is that milk production can be explained by BCS at calving; however, it is well established the main roles of the genetic merit and nutrition on milk production [12,25,26]. Based on previous reports and our records, our assumption was that cows in these small-scale farms in this region possess medium-high genetic merit for milk production [27,28] and that the nutrition is deficient [17,20]; therefore, we have discussed the results based on these assumptions. BCS at calving had a significant effect on milk production at 60 dim (*p* < 0.05, Figure 2a), while a statistical trend was observed for 305-d standardized milk production (*p* = 0.055, Figure 2b). In other production systems there has been a quadratic effect of BCS at calving on 60-d and 305-d milk production [12,29]. These studies have reported that the lower BCS at calving, the lower milk production due to reduced total energy (ingested + stored) available for milk production [12]. On the other hand, cows with higher BCS at calving present a reduced dry matter intake [30]. This leads to a greater mobilization of body energy reserves, a higher incidence of metabolic disorders, and lower milk production [30,31]. In the present study, the quadratic effect could not be evaluated because of the reduced number of thin and obese cows available. However, the strategy for the categorization by quartiles of the present study allowed one to identify the association between BCS at calving and 60-d and 305-d milk production. The highest proportion of cows in the lower quartile of 60-d and 305-d milk production had a BCS ≤ 2.5 at calving (48% in both). Conversely, the proportion of cows in the higher quartile of the distribution for 60-d milk production was higher for cows with a BCS ≥ 3.0 at calving (43%). These results were expected, since body energy reserves (reflected in the BCS) are a key factor in milk production [29,32]. On the other hand, in this dairy production system, cows with low BCS at calving (≤ 2.5, 36.6% in this study) are common, due to the inadequate daily energy intake of these cows [17,20]. Additionally, low BCS at calving (≤ 2.5) is one of the main risk factors associated with reproductive performance in small-scale dairy farms [10]. Therefore, we consider that more studies are needed to determine the genetic merit of cows in these dairy farms and to understand the partition of energy (energy available for production and reproduction), as well as to determine the incidence of health and metabolic disorders (clinical and subclinical) and the impact on profitability in cows with low BCS at calving with moderate levels of milk production.

Several studies have shown that multiple factors affect conception rate at first service and milk production in intensive dairy farms [12,13,14,15]. However, it was not known whether these factors impact reproductive and productive performance in cows in small-scale dairy farms. Here, the importance of BCS on conception rate and milk production in cows under small-scale dairy farms is reported. Additionally, the results highlight important interactions between the intervening factors [33], which suggests more precise management practices. It was also observed that small-scale dairy farms could represent a unique model for the study of the use of energy in cattle, where cows face constant underfeeding [17,20], produce suboptimal levels of milk (given the assumed medium-high milk production potential; [27,28]), and perform better reproductively than cows in intensive dairy farms [10,18]. Finally, these characteristics reinforce the idea that this production system possesses high potential to increase milk production and could be a strategic target to improve milk supply in the future [34].

## 4. Conclusions

It was concluded that, in small-scale dairy farms of western Mexico, high or low BCS at calving do not alter first-service conception rate in cows with ≥ 3 lactations. However, low BCS at calving increases first-service conception rate in first and second-lactation cows, which could be associated with lower metabolic stress as a result of lower milk production; the role of cows’ genetic merit for this response remains to be determined. Additionally, low BCS at calving was negatively associated with 60-d and 305-d milk production.

## Figures and Tables

**Table 1:** Descriptive statistics for body condition score, milk production, milk components, and days to first service in 24 small-scale dairy production farms in western Mexico (279 lactations).

Variable	Mean + SEM	Q1	Median	Q3
Body condition score at calving	2.7 ± 0.02	2.5	2.75	3
Body condition score at first service	2.6 ± 0.01	2.5	2.75	2.75
Change in body condition score	0.1 ± 0.02	0	0	0.25
Daily milk yield at 60 DIM (kg/d)	22.8 ± 0.46	17	22	28
305-d milk production (kg)	6688 ± 114	5450	6665	7920
305-d milk fat yield (kg)	192.7 ± 7.12	134	220	267
305-d milk protein yield (kg)	169.0 ± 6.06	122	193	234
Days postpartum to first service	84.2 ± 2.37	54	77	105

Q1, lower quartile; Q3, higher quartile; SEM, standard error mean; DIM, days in milk.

**Table 2:** Probability values and odds ratios between study variables and first-service conception rate; analysis of simple models.

Variable	*p*-Value	Categories	OR	95% CI
Lactation number	0.093 *	1	Ref.	-
		2	0.66	0.35–1.26
		3	0.52	0.24–1.12
		≥4	0.47	0.25–0.86
Body condition score at calving	0.325 *	Q1 (≤ 2.5)	Ref.	-
		Me (> 2.5–< 3.0)	0.88	0.50–1.55
		Q3 (≥ 3.0)	0.65	0.36–1.15
Body condition score at first service	0.048 *	Q1 (≤ 2.5)	Ref.	-
		Me Q3 (> 2.5)	1.62	1.01–2.61
Change in body condition score	0.890	Non-significant		
Daily milk yield at 60 DIM (kg/d)	0.070 *	Q1 (≤ 17)	Ref.	-
		Me (> 17–< 28)	1.09	0.62–1.94
		Q3 (≥ 28)	0.56	0.29–1.08
305-d milk yield	0.516	Non-significant		
305-d milk fat yield	0.934	Non-significant		
305-d milk protein yield	0.144 *	Q1 (≤ 122)	Ref.	-
		Me (> 122–< 234)	1.34	0.78–2.31
		Q3 (≥ 234)	0.73	0.38–1.41
Days postpartum to first service	0.965	Non-significant		

OR, odds ratio; 95% CI, 95% confidence interval; Me, values between Q1 and Q3; Ref., reference; -, 95% CI not calculated for being the reference; * level of significance for simple models was set at *p* < 0.35.

**Table 3:** Multiple models with non-collinear regressors for conception rate to first service.

Model	Regressors
1	Lactation number + BCS at calving + standardized protein
2	BCS at first service + daily milk yield at 60 DIM

BCS, body condition score.

**Table 4:** Results of logistic regression analysis for conception rate to first service; models 1 and 2.

Model	Variable	DF	Wald χ2	*p*-Value
1	Lactation number	3	5.878	0.118
	BCS at calving	2	1.459	0.482
	Lactation number and BCS at calving	6	10.755	0.096 *
2	BCS at first service	1	5.988	0.014 *
	Daily milk yield at 60 DIM	2	3.348	0.188
	BCS at first service and milk yield at 60 DIM	2	5.610	0.061 *

DF, degrees of freedom; * level of significance for multiple models was set at *p* < 0.1.
